# eHealth Literacy and Search Frequency in Relation to Objective Sleep Disorder Knowledge: Cross-Sectional Study

**DOI:** 10.2196/69588

**Published:** 2025-12-01

**Authors:** Jihyeon Oh, Christian Montag, Peter Johannes Schulz

**Affiliations:** 1 Faculty of Communication, Culture and Society, Università della Svizzera Italiana, Via G. Buffi 13 Lugano, Lugano, 6900, Switzerland, 41 586664724; 2 Communication and Media Research Center, Ewha Womans University, Seoul, Republic of Korea; 3 Centre for Cognitive and Brain Sciences, Institute of Collaborative Innovation, University of Macau, Macao SAR, China; 4 Department of Computer and Information Science, Faculty of Science and Technology, University of Macau, Macao SAR, China; 5 Department of Psychology, Faculty of Social Sciences, University of Macau, Macao SAR, China; 6 Wee Kim Wee School of Communication and Information/LKC School of Medicine, Nanyang Technological University, Singapore, Singapore; 7 Department of Communication and Media, Ewha Womans University, Seoul, Republic of Korea

**Keywords:** eHealth literacy, objective knowledge, search frequency, sleep disorders, digital health

## Abstract

**Background:**

The increasing use of the internet for health information has made eHealth literacy a critical factor in health knowledge acquisition and management. While eHealth literacy has been associated with positive health behaviors and knowledge in various contexts, its impact on disease-specific knowledge, particularly for sleep disorders, remains limited.

**Objective:**

This study aimed to examine the relationship between eHealth literacy and search frequency with objective knowledge about sleep disorders, and test whether the frequency of health information searches moderates the association between eHealth literacy and knowledge.

**Methods:**

An online survey was conducted with 266 adult participants. eHealth literacy was measured using the revised eHealth Literacy Scale, and the frequency of health information searches was assessed using a single-item scale. Objective knowledge about sleep disorders was evaluated using a newly developed scale tailored for this study. Hierarchical regression analysis was conducted to examine the main effects of eHealth literacy and search frequency on sleep disorder knowledge, while controlling for demographic variables such as age, gender, education, and past experience with sleep disorders. The interaction effect between eHealth literacy and frequency of health information searches on sleep disorder knowledge was further assessed using the PROCESS macro (model 1) to explore moderation effects.

**Results:**

Higher eHealth literacy was positively associated with greater objective knowledge about sleep disorders (*B*=0.27, SE 0.06, *P*<.001). Search frequency also showed a significant positive association with knowledge (*B*=0.24, SE 0.06; *P*<.001). In addition, the interaction between eHealth literacy and search frequency was significant (*B*=−0.61, SE 0.27; *P*=.03). Specifically, the positive link between eHealth literacy and knowledge was the strongest among individuals who searched less frequently and weakened as search frequency increased, becoming nonsignificant at the highest observed level of search frequency.

**Conclusions:**

eHealth literacy and search frequency were both linked to greater knowledge of sleep disorders. However, the strength of the literacy-knowledge association differed across levels of search frequency. The relationship was stronger when search frequency was low and weaker among frequent seekers. These findings suggest that eHealth literacy and information-seeking behaviors jointly shape factual knowledge. Supporting eHealth literacy together with guidance on efficient information use may help patients acquire more accurate health knowledge. This study also introduces a newly developed scale for assessing objective knowledge about sleep disorders, providing a foundation for consistent evaluation of disease-specific knowledge.

## Introduction

### Background

Sleep disorders are a significant public health concern, with about two-thirds of adults worldwide reporting at least one sleep-related problem [1]. Their prevalence has been confirmed not only among the general adult population [2] but also in specific groups such as nursing students [3] and cancer survivors [4]. The consequences extend beyond physical health, as poor sleep has been consistently linked to adverse mental health outcomes [5] and overall diminished well-being [6]. Conditions such as insomnia, sleep apnea, and restless legs syndrome affect individuals across different age groups, significantly reducing quality of life [7]. While severe cases may require medical treatment, early intervention through lifestyle adjustments and access to reliable information is crucial for mitigating symptoms in their initial stages [8]. If left unaddressed, the chronic nature of sleep disorders can result in long-term health problems, underscoring the need for accessible and trustworthy health information.

As many individuals are unaware of the early signs of these conditions, they often turn to the internet for information, which may not always be accurate or trustworthy [9]. This points to the importance of eHealth literacy—the ability to find, understand, and apply reliable health information found online [10]. eHealth literacy has been conceptualized as a multidimensional construct extending traditional notions of health literacy to the digital environment, encompassing skills to navigate, evaluate, and use online health resources effectively [11]. However, individuals seeking health information online encounter challenges such as information overload [12] and exposure to health misinformation [13], highlighting why eHealth literacy remains crucial for the effective use of online health resources [14].

The most widely used instrument, eHealth Literacy Scale (eHEALS), is based on self-reports and does not cover functional, communicative, or critical skills [15]. To address these limitations, alternative instruments have been developed, such as the Transactional Model of eHealth Literacy, which outlines functional, communicative, critical, and translational dimensions of eHealth literacy [16], and the CoV-eHEALS, which was validated during the COVID-19 pandemic but also revealed the tendency of self-reports to overestimate actual ability [17]. Nevertheless, most studies continue to rely on self-reported measures, leaving the link between perceived ability and actual knowledge uncertain [15]. Reviews have emphasized the importance of examining how eHealth literacy relates to objective knowledge outcomes [14].

Search frequency has been shown to exert an independent influence on knowledge acquisition. On the one hand, more frequent searching can increase awareness and exposure to health information [18,19]. On the other hand, it may also lead to information overload and conflicting messages, which can reduce factual accuracy [20,21]. This dual nature suggests that search frequency itself functions as a predictor of knowledge outcomes, beyond the role of eHealth literacy. Prior research also indicates that eHealth literacy predicts positive health behaviors even after controlling for search frequency [18], underscoring the importance of considering search frequency and literacy as distinct explanatory factors.

In addition, information-seeking behaviors may influence how eHealth literacy translates into knowledge. While prior work has typically examined literacy and information seeking as separate predictors [18] or emphasized their independent contributions [22], less attention has been paid to their potential interaction. This gap highlights the need to examine whether the effect of eHealth literacy on knowledge varies depending on search frequency. In the context of sleep disorders, where accurate knowledge is essential but often limited, such an interplay may be particularly consequential.

Research on other conditions supports this view. eHealth literacy has been linked to knowledge in chronic obstructive pulmonary disease [22], colorectal cancer [23], and COVID-19 [17]. However, such evidence is scarce in the context of sleep. The concept of sleep health literacy emphasizes the competencies required to recognize and manage sleep problems [24]. Building on this, recent studies have introduced validated measurement tools for sleep health literacy [25]. Yet, awareness of sleep disorders remains low even among medical professionals [26], and education has been shown to improve knowledge without necessarily enhancing sleep quality [27]. These observations indicate that knowledge about sleep disorders is both important and underexplored. Thus, there is a need to examine both the independent and combined effects of eHealth literacy and search frequency in shaping sleep disorder knowledge.

### Objectives

**Figure 1.**
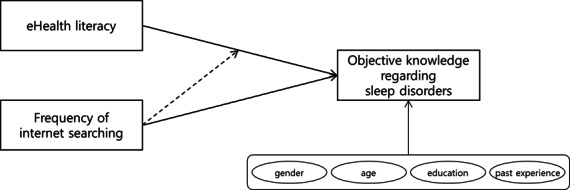
Hypothesized research model.

This study addresses this gap by examining how eHealth literacy and search frequency jointly influence objective knowledge about sleep disorders. By testing both their independent and interactive contributions, the study extends prior work that has typically considered these factors in isolation, situating the analysis in the understudied context of sleep disorders. Figure 1 illustrates the hypothesized research model.

Our hypotheses were as follows:

- H1: eHealth literacy will be positively associated with objective knowledge about sleep disorders.
- H2: Search frequency will be positively associated with objective knowledge about sleep disorders.
- H3: The association between eHealth literacy and objective knowledge will be moderated by the frequency of health information searches.

- H2: Search frequency will be positively associated with objective knowledge about sleep disorders.
- H3: The association between eHealth literacy and objective knowledge will be moderated by the frequency of health information searches.

## Methods

### Participants

The study employed a cross-sectional online survey design and initially recruited 280 participants from psychology classes at Ulm University in Ulm, Germany, using a convenience sampling approach. Eligibility criteria required participants to be at least 18 years old and proficient in the German language. After excluding 14 participants, the data from 266 respondents were used for analysis. Specifically, 9 participants were excluded because they were not fluent in German, and 5 participants were excluded for not completing the survey in a serious manner, as indicated by inconsistent or patterned responses. A sensitivity power analysis using G*Power (*F* tests, multiple regression: *R*^2^ increase; *α*=.05, 1–*β*=.80, 1 tested predictor, total predictors=7) indicated that with 266 participants, the study was sufficiently powered to detect a small incremental effect (*f*^2^=0.03).

### Ethical Considerations

The study was reviewed by the ethics committee of Ulm University, which determined that detailed ethical approval was not necessary. Participation was voluntary, and all respondents provided informed consent before beginning the survey. Data were collected anonymously through an online survey tool (Survey Coder Tool by Christopher Kannen [28]), and no personally identifying information was retained. Participants received course credit as compensation for their involvement. Participants were also informed that their anonymized data could be shared for research purposes following publication. The study design and reporting followed the CHERRIES (Checklist for Reporting Results of Internet E-Surveys; Checklist 1).

### Measurements

#### eHealth Literacy

The level of eHealth literacy was assessed using the revised eHEALS. This scale is based on the original eHEALS items developed by Norman and Skinner [10], with additional items from the eHEALS-E questionnaire [11]. The German version of the scale was applied (see Multimedia Appendix 1), with all items translated into German (2 independent bilingual scientists performed a back-and-forth translation). The eHEALS consists of 8 questions, each rated on a 5-point Likert scale ranging from 1 (strongly disagree) to 5 (strongly agree), with a total score ranging from 8 to 40, measuring participants’ perceived eHealth literacy. The validity of the revised version of eHEALS was confirmed through a confirmatory factor analysis. The analysis of the 8-item model indicated a good fit for the proposed model (*χ*^2^_7_=4.728, *P*=.693, comparative fit index=1.000, Tucker-Lewis index=1.000, root mean square error of approximation=0.000, standardized root mean square residual=0.013). The internal consistency of the test was confirmed using McDonald’s omega coefficient (*ω*=0.854). The specific items of this eHEALS were as follows:

1. I know how to find helpful health information on the internet.
2. I know how to use the internet to answer my health questions.
3. I know what health resources are available on the internet.
4. I know where to find helpful health resources on the internet.
5. I know how to use the health information I find on the internet to help me.
6. I have the skills I need to evaluate the health resources I find on the internet.
7. I can tell high-quality from low-quality health resources on the internet.
8. I feel confident in using information from the internet to make health decisions.

#### Objective Knowledge Regarding Sleep Disorders

Objective knowledge about sleep disorders was measured using a 15-item scale, adapted from a previous study [29]. A total of 15 items were initially measured on a 7-point scale (1=Sure this is incorrect, 7=Sure this is correct) and later dichotomized into correct (1) and incorrect (0) responses for hypothesis testing. The German version of the scale was applied (see Multimedia Appendix 2), with all items translated into German (again, 2 bilingual scientists performed the back-and-forth translation process, see above). The specific items are listed below, with the correct answers indicated in parentheses (T=true, F=false). The total score ranged from 0 to 15, with higher scores reflecting greater factual knowledge of sleep disorders.

1. Drinking 3 standard glasses of alcohol has no effect on sleep. (F)
2. Taking a prescription sleep medication can cause stomach problems like nausea and gas. (T)
3. Smoking more than 1 pack of cigarettes a day has no effect on sleep. (F)
4. Leaving a light on benefits sleep. (F)
5. Going to bed thirsty has an effect on sleep. (T)
6. If you cannot fall asleep in 20 minutes, you should get out of bed and try again later. (T)
7. Prescription sleep medications are designed to be taken for a long period of time. (F)
8. Going to bed at the same time each night disrupts sleep. (F)
9. You should spend 2 hours longer in bed than you need for sleep to give yourself the best opportunity. (F)
10. Consuming food, beverages, or medications containing caffeine has no effect on sleep. (F)
11. Regular exercise at least 4 hours before going to bed benefits sleep. (T)
12. Prescription sleep medications should be taken just before going to bed. (T)
13. If you wake during the night and cannot fall back to sleep within 20 min, you should stay in bed and try harder. (F)
14. Stress and anxiety can make it harder to fall asleep but do not affect the length of sleep. (F)
15. Newer prescription sleep medications (nonbenzodiazepines) are safer because they do not lead to dependence. (F)

#### Frequency of Internet Searching

The frequency of internet searches for health information was assessed based on the daily number of searches conducted by the participants. This frequency was measured using a single-item scale (also presented in the German language). Respondents indicated their agreement with the following statements: “Have you searched on the internet for information on your health, health problems, or medical treatments in the last 6 months? (Haben Sie in den letzten sechs Monaten im Internet nach Informationen zu Ihrer Gesundheit, Gesundheitsproblemen oder medizinischen Behandlungen gesucht?)” The item was measured on a 3-point Likert scale (1=None, 2=1 or 2 times, 3=More than twice). Previous studies have identified a positive association between eHealth literacy and the frequency of internet searches [18,19]. Based on the previous studies, in this study, we used the frequency of internet searching as a moderating variable to examine how it influences the relationship between eHealth literacy and objective knowledge about sleep disorders.

#### Covariates

In addition to the above endogenous variables, we also controlled for our participants’ age, gender, education level, and past experience with sleep disorders as exogenous variables to make sure that the sample represents the population.

### Statistical Analysis

To test H1 and H2, hierarchical multiple regression analysis was conducted with objective knowledge as the dependent variable. In step 1, demographic variables (age, gender, and education) were entered as controls. In step 2, past experience with sleep disorders was added. In step 3, eHealth literacy was included, and in step 4, search frequency was added. Continuous predictors were mean-centered prior to these regression analyses. To test H3, the interaction between eHealth literacy and search frequency was examined using the PROCESS macro for SPSS (model 1; Hayes [30]) with 5000 bootstrap samples [30]. This approach estimated the conditional effects of eHealth literacy on knowledge at different levels of search frequency. All analyses were performed with SPSS Statistics (version 26; IBM Corp.).

## Results

### Validation of the Objective Knowledge Regarding Sleep Disorders

To evaluate the properties of the adapted sleep knowledge scale, item- and scale-level analyses were conducted. Item-total correlations were generally low, which is expected in factual knowledge tests because each item represents an independent piece of information. Sampling adequacy was acceptable (Kaiser-Meyer-Olkin=0.667), and Bartlett test indicated that the correlation matrix was factorable (*χ*^2^_105_=369.25; *P*<.001). An exploratory factor analysis using principal axis factoring with oblimin rotation suggested limited unidimensionality: although 6 components had eigenvalues greater than 1 (first eigenvalue=2.59), the first extracted factor explained only 13.14% of the variance, and item loadings were widely dispersed. Internal consistency based on a single-factor model was also low (*ω*=0.20), consistent with the expectation that the scale functions as an index of heterogeneous factual knowledge rather than a unidimensional construct.

Evidence for validity was supported by positive correlations of the knowledge score with eHealth literacy (*r*=0.30; *P*<.001) and with recent general online health information seeking (*r*=0.27; *P*<.001). In contrast, neither sleep problem experience (*r*=−0.03; *P*=.67) nor sleep-specific information seeking (*r*=−0.01; *P*=.89) was significantly related to knowledge.

Known-groups validity was further supported by higher knowledge scores among participants who had searched for health information in the past 6 months compared with those who had not (mean difference=1.27, *P*=.004 for none vs once or twice; mean difference=1.84, *P*<.001 for none vs more than twice; Bonferroni-adjusted). No significant group differences were observed for sleep problem experience (*F*_3,262_=0.78; *P*=.50) or for sleep-specific searches (*F*_2,263_=2.10; *P*=.13). Taken together, these findings indicate that the adapted scale captures factual accuracy across diverse content areas and is appropriate for use as a knowledge index in this study.

### Sample Characteristics

The study sample consisted of 266 participants, of whom 229 (86.1%) were female and 37 (13.9%) were male. The mean age of the respondents was 21.78 (SD 3.35; range 18‐52) years. Most (n=217, 81.6%) participants were in the 20‐29 years age range. A smaller proportion (n=42, 15.8%) were younger than 20 years, 2.4% (n=6) were aged 30‐39 years, and 0.4% (n=1) were 40 years and older. Regarding educational attainment, the majority (n=242, 91.0%) held a high school diploma. Other levels included intermediate technical qualifications (n=4, 1.5%), University of Applied Sciences degrees (n=3, 1.1%), and university degrees (n=16, 6.0%). Only 1 (0.4%) participant reported a secondary school leaving certificate. All participants were native German speakers (n=266, 100%). These demographic characteristics are summarized in Table 1.

**Table 1.** Participants’ demographics (N=266).

Characteristic	Participants, n (%)
Gender	
Male	37 (13.9)
Female	229 (86.1)
Age (y)	
<20	42 (15.8)
20‐29	217 (81.6)
30‐39	6 (2.4)
≥40	1 (0.4)
Education achievement	
Secondary school leaving certificate	1 (0.4)
Intermediate technical qualification	4 (1.5)
High school diploma	242 (91)
University of applied sciences degree	3 (1.1)
University degree	16 (6)
Native German	
Yes	266 (100)
No	0 (0)

Prior to analyzing the hypothesized model, descriptive statistics and Pearson correlations were computed (Table 2).

**Table 2.** Means, standard deviations, and correlations of all variables^a^.

	1^b^	2	3	4	5	6	7
Age	1						
Gender	−0.10	1					
Education	0.14	0.04	1				
Past experience	0.03	0.04	0.05	1			
eHealth literacy	0.07	−0.02	0.06	−0.04	1		
Search frequency	−0.03	0.04	0.04	0.02	0.08	1	
Objective knowledge	−0.02	0.02	0.09	−0.03	0.30	0.27	1
Mean (SD)	21.78 (3.35)	1.86 (.35)	5.11 (.52)	2.50 (.99)	3.68 (.64)	2.32 (.70)	11.24 (2.14)

a Values are Pearson correlation coefficients. Correlations of age with education (*r*=0.14; *P*=.02), eHealth literacy with objective knowledge (*r*=0.30; *P*<.001), and search frequency with objective knowledge (*r*=0.27; *P*<.001) were significant; all other correlations were nonsignificant.

b Numbers (1-7) refer to the variable labels listed in the leftmost column (Age to Objective knowledge).

### Main Association Analysis

To test H1 and H2, we conducted hierarchical multiple regression analyses with objective knowledge as the dependent variable. Table 3 summarizes the results.

**Table 3.** Results of hierarchical multiple regression analyses on objective knowledge (N=266)^a^.

	Objective knowledge				
	*B* ^b^	SE	*ß* ^c^	*t* test (*df*)	*P* value
Step 1^d^					
Age	–0.03	0.06	–0.03	–0.49 (262)	.62
Gender	–0.01	0.06	–0.01	–0.14 (262)	.89
Education	0.09	0.06	0.09	1.47 (262)	.14
Step 2^e^					
Age (y)	–0.03	0.06	–0.03	–0.48 (261)	.63
Gender	–0.01	0.06	–0.01	–0.15 (261)	.88
Education	0.09	0.06	0.09	1.49 (261)	.14
Past experience	–0.03	0.06	–0.03	–0.49 (261)	.63
Step 3^f^					
Age	–0.05	0.06	–0.05	–0.78 (260)	.44
Gender	–0.01	0.06	–0.01	–0.23 (260)	.82
Education	0.07	0.06	0.08	1.28 (260)	.20
Past experience	–0.02	0.06	–0.02	–0.29 (260)	.77
eHealth literacy	0.29	0.06	0.30	4.97 (260)	<.001
Step 4^g^					
Age	–0.04	0.06	–0.04	–0.63 (259)	—^h^
Gender	–0.01	0.06	–0.01	–0.08 (259)	—
Education	0.07	0.06	0.06	1.16 (259)	—
Past experience	–0.02	0.06	–0.02	–0.39 (259)	—
eHealth literacy	0.27	0.06	0.27	4.75 (259)	<.001
Search frequency	0.24	0.06	0.24	4.20 (259)	<.001

a Gender coded as female=0, male=1. Δ*R*2 values correspond to variance explained by each step.

b *B*: unstandardized coefficient.

c *β*: standardized coefficient.

d Model summary: *P*=.51; *F*_3,262_=0.768; Δ*R*2=0.009.

e Model summary: *P*=.64; *F*_4,261_=0.634; Δ*R*2=0.001.

f Model summary: *P*<.001; *F*_5,260_=5.488; Δ*R*2=0.086.

g Model summary: *P*<.001; *F*_6,259_=7.801; Δ*R*2=0.058.

h Not applicable.

In step 1, demographic variables including age, gender, and education were entered into the model. Age, gender, and education were not significant predictors of objective knowledge. This model accounted for 0.9% of the variance (Δ*R*^2^=0.009; *F*_3,262_=0.768; *P*=.51).

In step 2, past experience with sleep disorders was added, but it did not significantly predict objective knowledge. This step explained an additional 0.1% of the variance (Δ*R*^2^=0.001; *F*_4,261_=0.634; *P*=.64).

In step 3, we added eHealth literacy to the model. eHealth literacy emerged as a significant predictor (*B*=0.29, SE 0.06; *t*_260_=4.97; *P*<.001), explaining an additional 8.6% of the variance (Δ*R*^2^=0.086; *F*_5,260_=5.488; *P*<.001). Higher eHealth literacy was associated with greater objective knowledge. However, age, gender, education, and past experience were nonsignificant.

In step 4, the search frequency was entered. Search frequency emerged as an additional significant predictor (*B*=0.24, SE 0.06; *t*_259_=4.20; *P*<.001), accounting for an additional 5.8% of the variance (Δ*R*^2^=0.058; *F*_6,259_=7.801; *P*<.001). The overall model explained 15.3% of the variance (*R*^2^=0.153).

In sum, these findings indicate that eHealth literacy and search frequency both contribute significantly to objective knowledge about sleep disorders, even after accounting for demographic factors and past experience with sleep disorders.

### Moderation Analysis

The main effect of eHealth literacy on objective knowledge about sleep disorders was significant, with higher eHealth literacy predicting greater knowledge (*B*=2.33, SE 0.66; *t*_259_=3.56; *P*<.001; 95% CI 1.04-3.62; see Table 4). Similarly, more frequent health information searches were associated with higher objective knowledge (*B*=3.00, SE 1.02; *t*_259_=2.94; *P*=.004; 95% CI 0.99-5.00). The interaction between eHealth literacy and health information search frequency was also significant (*B*=−0.61, SE 0.27; *t*_259_=−2.24; *P*=.03; 95% CI −1.15 to −0.07), indicating a moderation effect.

**Table 4.** Moderated effect: search frequency as a moderator in the association between eHealth literacy and objective knowledge (N=266)^a^.

Predictor^b^	*B*	SE	*t *test (*df*)	*P* value	95% CI
eHealth literacy	2.33	0.66	3.56 (259)	<.001	1.04 to 3.62
Search frequency	3.00	1.02	2.94 (259)	.004	0.99 to 5.00
eHealth literacy × search frequency	–0.61	0.27	–2.24 (259)	.03	–1.15 to –0.07

a Covariates (age, gender, and past experience with sleep disorders) were included in the model.

b Model summary: *R*2=0.165; *F*_6,259_=8.52; *P*<.001.

Conditional effects showed that when search frequency was low (−1 SD), the positive effect of eHealth literacy on knowledge was strong (*B*=1.35, SE 0.27; *t*_259_=5.04; *P*<.001; 95% CI 0.82 to 1.87). At the mean level of search frequency, the effect remained significant but weaker (*B*=0.92, SE 0.19; *t*_259_=4.80; *P*<.001; 95% CI 0.54-1.30). At the highest observed level of search frequency, the effect was no longer significant (*B*=0.50, SE 0.27; *t*_259_=1.86; *P*=.06; 95% CI −0.03 to 1.03). These findings indicate that the contribution of eHealth literacy to knowledge acquisition depends on search frequency, with the effect attenuating as search frequency increases. The overall model was significant (*R*^2^=0.165; *F*_6,259_=8.52; *P*<.001). These conditional effects are summarized in Table 5.

**Table 5.** Conditional effects of eHealth literacy on objective knowledge at values of search frequency^a^.

Search frequency level	*B*	SE	*t* test (*df*)	*P* value	95% CI
Low (–1 SD)	1.35	0.27	5.04 (259)	<.001	0.82 to 1.87
Mean	0.92	0.19	4.80 (259)	<.001	0.54 to 1.30
High (maximum observed)	0.50	0.27	1.86 (259)	.06	–0.03 to 1.03

a Conditional effects estimated with PROCESS Model 1 (5000 bootstrap samples).

**Figure 2.**
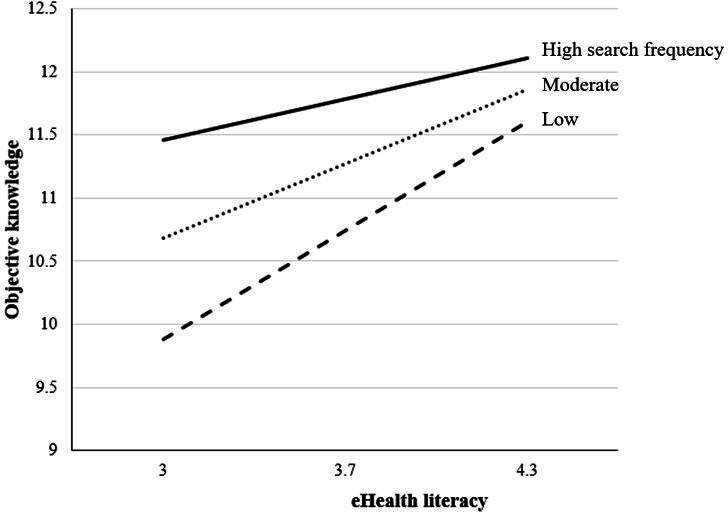
Objective knowledge as a function of eHealth literacy and search frequency.

These results are illustrated in Figure 2, which depicts the interaction effects between eHealth literacy and health information search frequency on objective knowledge.

## Discussion

### Principal Results

This study investigated how eHealth literacy and the frequency of health information searches contribute to knowledge about sleep disorders. The results indicated positive main effects of both eHealth literacy (H1) and search frequency (H2), as well as a moderation effect showing that the contribution of literacy was conditioned by search frequency (H3).

### Comparison With Previous Work

Consistent with H1, higher eHealth literacy was positively associated with greater knowledge about sleep disorders. Prior research has shown that literacy enhances the ability to evaluate online health information accurately [31] and contributes to more reliable knowledge in disease-specific contexts such as COVID-19 [17]. Similar associations have also been reported in colorectal cancer [23,32].

In line with H2, search frequency itself was positively associated with knowledge outcomes. Studies have indicated that active searching increases awareness and factual accuracy [18,19]. At the same time, frequent searching can expose individuals to information overload or conflicting messages, which may reduce accuracy [20,21]. These findings suggest that the effects of search frequency are not uniform across contexts.

Most importantly, H3 was supported. The positive association between eHealth literacy and knowledge was the strongest when search frequency was low, whereas the effect diminished and became nonsignificant at higher levels of searching. This pattern demonstrates that eHealth literacy and information-seeking behavior interact to determine knowledge outcomes rather than exerting independent effects. Previous studies, however, have primarily examined the independent effects of literacy [18] or of search behavior [18,19], without considering how they may interact. Our findings highlight that treating literacy and search in isolation may overlook the ways in which cognitive capacities and behavioral patterns jointly shape knowledge outcomes. The observed attenuation of literacy’s effect at higher search frequencies may be explained by multiple mechanisms, including information overload that reduces the marginal value of repeated searching [19] and variability in the quality of retrieved information [31]. Together, these results underscore that eHealth literacy and information-seeking behavior are interdependent factors that jointly shape how effectively individuals acquire accurate health knowledge.

Finally, this study extends eHealth literacy research to the underexplored context of sleep disorders. Few studies have examined sleep-specific knowledge in relation to literacy [24,25]. Intervention studies have also yielded mixed outcomes, with awareness and knowledge improving but effects on sleep quality being less consistent [27]. By developing and validating a new measure of objective knowledge about sleep disorders, this study addresses this gap and provides a methodological contribution to advancing sleep health literacy research.

### Limitations, Implications, and Future Directions

Beyond these findings, several limitations should be acknowledged. First, the sample size, while sufficient for the analyses conducted, may limit the generalizability of the findings. The sample was heavily skewed toward young, female psychology students (N=266; mean age=21.78, SD 3.35; n=229, 86.1% female), which constrains external validity. Prior studies have shown that eHealth literacy and information processing can vary across age [33] and education levels [18,34], suggesting that the present findings may not generalize to broader populations. The restricted age variance may also help explain why demographic effects, such as age, were not significant in this study. Future research should seek to include a larger, more diverse population to strengthen the external validity of these results.

Second, while this study successfully demonstrated the association between eHealth literacy and actual knowledge levels, it did not account for the outcomes resulting from actual behavior. Future research should examine whether and how these search activities lead to increased knowledge, and more importantly, how these processes occur. This could involve investigating the quality of information accessed, the strategies used to evaluate and apply this information, and the extent to which these factors contribute to knowledge acquisition and behavior change.

Third, the study highlighted the interaction pattern of search behavior on the relationship between eHealth literacy and objective knowledge. However, it did not delve into the underlying causes of this search behavior. Future research should consider various factors related to media search behavior, such as motivation, accessibility, and past experiences, to better understand the background of these actions. By exploring these variables, researchers can gain insights into what drives individuals to engage in frequent health information searches and how these behaviors interact with eHealth literacy to influence knowledge outcomes.

Lastly, the measure of health information search frequency was self-reported, which introduces potential biases in reporting. Participants may overestimate or underestimate their search behavior, which could influence the accuracy of the results. Objective measures of search behavior, such as tracking actual online searches, would provide a more precise understanding of how frequently individuals seek out health information and how that correlates with knowledge acquisition.

Beyond methodological limitations, the findings also carry theoretical and practical implications. Theoretically, this study contributes to eHealth literacy research by clarifying that the effect of literacy is most evident when search frequency is low, and by demonstrating the value of integrating a behavioral factor as a moderator. It also offers a methodological contribution through the development of a disease-specific objective knowledge scale for sleep disorders. This scale can serve as a useful tool for evaluating the outcomes of literacy-based interventions and for assessing knowledge of sleep disorders in future research. Practically, the results point to the value of strengthening literacy among individuals who search less frequently, while digital platforms may support low-literacy users by embedding credibility cues or sleep-education modules. Taken together, these insights suggest that literacy enhancement efforts should be accompanied by training on critical information management, which can inform digital health education and patient-support initiatives. Such strategies can provide useful guidance for educators, clinicians, and public health agencies seeking to improve sleep-related health knowledge.

### Conclusion

This study examined how eHealth literacy and health information seeking jointly influence knowledge acquisition. We found that higher eHealth literacy was associated with greater sleep disorder knowledge, and more frequent health information searches were also positively related to knowledge. However, the effect of eHealth literacy was varied by search frequency. The positive association was evident among individuals who searched less often, but it diminished at higher levels of search activity. These findings highlight that the role of eHealth literacy is not uniform and depends on behavioral patterns of information seeking. Understanding this interaction clarifies how cognitive capacities and behaviors jointly influence knowledge acquisition and has implications for digital health education, suggesting that literacy support may be more effective when combined with strategies for managing online information. Overall, the study contributes by clarifying the conditions under which eHealth literacy exerts its strongest effects and by extending research to the context of sleep disorders with a newly developed objective knowledge scale. This scale can be used to assess knowledge of sleep disorders and to guide literacy-based health education.

## Supplementary material

10.2196/69588 Multimedia Appendix 1 eHealth literacy scale (German version).

10.2196/69588 Multimedia Appendix 2 Objective knowledge regarding sleep problems scale (German version).

10.2196/69588 Checklist 1 CHERRIES checklist.

## Abbreviations

CHERRIES: Checklist for Reporting Results of Internet E-Surveys
eHEALS: eHealth Literacy Scale
