# Supplementary material for: eHealth Literacy and Search Frequency in Relation to Objective Sleep Disorder Knowledge: Cross-Sectional Study
Source: J Med Internet Res. 2025 Dec 1;27:e69588. doi: 10.2196/69588 (PMC12670055; doi:10.2196/69588)
Supplement: Multimedia Appendix 1 [file jmir-v27-e69588-s001.docx]

1. Ich weiß, wie ich Internetseiten mit hilfreichen Gesundheitsinformationen finden kann.
2. Ich weiß, wie ich das Internet nutzen kann, um Antworten auf meine Gesundheitsfragen zu erhalten.
3. Ich weiß, welche Seiten mit Gesundheitsinformationen im Internet verfügbar sind.
4. Ich weiß, wo ich im Internet hilfreiche Gesundheitsinformationen finden kann.
5. Ich weiß Gesundheitsinformationen aus dem Internet so zu nutzen, dass sie mir weiterhelfen.
6. Ich bin in der Lage, Internetseiten mit Gesundheitsinformationen kritisch zu bewerten.
7. Ich kann zwischen vertrauenswürdigen und fragwürdigen Internetseiten mit Gesundheitsinformationen unterscheiden.
8. Ich fühle mich sicher darin, Informationen aus dem Internet zu nutzen, um Entscheidungen in Bezug auf meine Gesundheit zu treffen.
