# Supplementary material for: eHealth Literacy and Search Frequency in Relation to Objective Sleep Disorder Knowledge: Cross-Sectional Study
Source: J Med Internet Res. 2025 Dec 1;27:e69588. doi: 10.2196/69588 (PMC12670055; doi:10.2196/69588)
Supplement: Multimedia Appendix 2 [file jmir-v27-e69588-s002.docx]

1. Das Trinken von drei Standardgläsern Alkohol hat keine Auswirkungen auf den Schla
2. Die Einnahme eines verschreibungspflichtigen Schlafmittels kann Magenprobleme wie Übelkeit und Blähungen verursachen.
3. Das Rauchen von mehr als einer Schachtel Zigaretten pro Tag hat keinen Einfluss auf den Schlaf.
4. Das Einschalten eines Lichts fördert den Schlaf.
5. Durstig ins Bett zu gehen, hat Auswirkungen auf den Schlaf.
6. Wenn Sie nicht innerhalb von 20 Minuten einschlafen können, sollten Sie aufstehen und es später erneut versuchen.
7. Verschreibungspflichtige Schlafmedikamente sind darauf ausgelegt, über einen längeren Zeitraum eingenommen zu werden.
8. Wenn man jeden Tag zur selben Zeit ins Bett geht, stört das den Schlaf.
9. Um gut zu schlafen, sollten Sie 2 Stunden länger im Bett verbringen als Sie für den Schlaf benötigen.
10. Das Verzehren von Lebensmitteln, Getränken oder Medikamenten mit Koffein hat keine Auswirkungen auf den Schlaf.
11. Regelmäßige körperliche Aktivität mindestens 4 Stunden vor dem Schlafengehen fördert den Schlaf.
12. Verschreibungspflichtige Schlafmedikamente sollten kurz vor dem Zubettgehen eingenommen werden.
13. Wenn Sie während der Nacht aufwachen und innerhalb von 20 Minuten nicht wieder einschlafen können, sollten Sie im Bett bleiben und sich mehr anstrengen.
14. Stress und Angst können es schwieriger machen, einzuschlafen, beeinflussen jedoch nicht die Schlafdauer.
15. Neuere verschreibungspflichtige Schlafmedikamente (Nicht-Benzodiazepine) sind sicherer, weil sie nicht zu Abhängigkeit führen.
