# Supplementary material for: eHealth Literacy and Search Frequency in Relation to Objective Sleep Disorder Knowledge: Cross-Sectional Study
Source: J Med Internet Res. 2025 Dec 1;27:e69588. doi: 10.2196/69588 (PMC12670055; doi:10.2196/69588)
Supplement: Checklist 1 [file jmir-v27-e69588-s003.docx]

| **Category** | **Item** | **Reported in manuscript** |
| --- | --- | --- |
| Design | Survey type/tool | Cross-sectional online survey using Survey Coder Tool (ckannen.com) |
| Ethics | IRB approval and consent | Reviewed by Ulm University ethics committee (detailed approval not required); electronic informed consent obtained |
| Recruitment | Open/closed survey, method | Closed survey; recruited from psychology classes at Ulm University |
| Participants | Inclusion/exclusion criteria | ≥18 years, fluent in German; exclusions: 9 not fluent, 5 careless responses |
| Sample size | Numbers invited, analyzed | 280 invited, 266 analyzed |
| Completion rate | Started, completed | 280 started; 266 completed (14 excluded for quality) |
| Preventing duplicates | Methods | One submission per participant; screened for patterned responses |
| Incentives | Compensation | Course credit |
| Data protection | Anonymity/security | Anonymous responses, no personal identifiers |
| Randomization / Adaptive questioning | Logic | None |
| Mandatory items | Required questions | All items were mandatory |
| Missing data | Handling | Incomplete or invalid responses excluded |
